# CRED9: a differentially expressed elncRNA regulates expression of transcription factor CEBPA

**DOI:** 10.1261/rna.078752.121

**Published:** 2021-08

**Authors:** Ryan L. Setten, Pritsana Chomchan, Elizabeth W. Epps, John C. Burnett, John J. Rossi

**Affiliations:** 1 Department of Molecular and Cellular Biology, Beckman Research Institute of City of Hope, Duarte, California 91010, USA; 2 Irell & Manella Graduate School of Biological Sciences, Beckman Research Institute of City of Hope, Duarte, California 91010, USA; 3 Center for Gene Therapy, Beckman Research Institute of City of Hope, Duarte, California 91010, USA

**Keywords:** enhancer RNA, elncRNA, enhancer, transcription factor, CEBPA, H3K27ac

## Abstract

Enhancer RNAs (eRNA) are noncoding transcripts produced from active enhancers and have potential gene regulatory function. CCAAT enhancer-binding protein alpha (CEBPA) is a transcription factor generally involved in metabolism, cell cycle inhibition, hematopoiesis, adipogenesis, hepatogenesis, and is associated with tumorigenesis. In this study, we demonstrate that an enhancer-associated long noncoding RNA (elncRNA), transcribed from an enhancer located 9 kb downstream from the transcriptional start site (TSS) of CEBPA, positively regulates the expression of CEBPA. As a result, we named this elncRNA “CEBPA regulatory elncRNA downstream 9 kb” or “CRED9.” CRED9 expression level positively correlates with CEBPA mRNA expression across multiple cell lines as detected by RT droplet digital PCR. Knockdown of CRED9 resulted in a reduction of CEBPA mRNA expression in Hep3B cells. Additionally, CRED9 knockdown in Hep3B and HepG2 cells resulted in lower CEBPA protein expression. We also found that knockdown of CRED9 in Hep3B cells caused a 57.8% reduction in H3K27ac levels at the +9 kb *CEBPA* enhancer. H3K27ac has previously been described as a marker of active enhancers. Taken together, the evidence presented here supports a previously proposed model whereby, in some contexts, eRNA transcripts are necessary to amplify and maintain H3K27ac levels at a given enhancer. Ultimately, this study adds to the growing body of evidence that elncRNA transcripts have important roles in enhancer function and gene regulation.

## INTRODUCTION

Enhancers are directionally independent DNA-based regulatory elements that, when active, augment transcription from a distally associated promoter. During enhancer activation, specific transcription factors bind to response elements encoded in enhancer DNA. Enhancer activation is thought to precede gene promoter activation in most cases. In this model, enhancers become activated by pioneer transcription factors (e.g., FOXO1), which are capable of binding to heterochromatin resulting in a less condensed DNA state. This state affords binding of lineage-specific factors like CEBPA, which further open up chromatin, and allow additional transcription factors or coactivators to bind. RNA polymerase II (Pol II) and other factors involved in transcription are also loaded onto enhancers, which result in the production of enhancer eRNAs (eRNAs) (Li et al. 2016). Looping factors bring enhancers and promoter(s) into contact with one another. Finally, transcription factors bound to an enhancer and promoter, together with coactivator proteins, induce transcription of the target promoter (Arner et al. 2015; Li et al. 2016).

eRNAs represent a subclass of long noncoding RNAs (lncRNA) that are transcribed from active enhancers (De Santa et al. 2010; Kim et al. 2010; Li et al. 2016; Arnold et al. 2020). Studies show that expression of eRNAs, along with histone H3K27ac and a high H3K4me1 to H3K4me3 ratio, is strongly predictive of enhancer activity in a given cell type (Barski et al. 2007; Creyghton et al. 2010; Melgar et al. 2011). eRNAs can be further categorized into bidirectionally transcribed 0.5–2 kb nonpolyadenylated transcripts and the less common unidirectionally transcribed, typically >4 kb and polyadenylated, transcripts referred to here and in other studies as enhancer-associated long noncoding RNAs (elncRNAs) (Koch et al. 2011). The mechanisms by which eRNAs carry out their function are still being determined. As a general example, nascently transcribed eRNAs have been demonstrated to stimulate HAT activity of CBP/p300 by binding to an RNA-binding region in the HAT domain of CBP (Bose et al. 2017). Stimulation of HAT activity leads to a local increase in acetylation of H3K27 and possibly to acetylation of other locally bound proteins. Less generally, individual eRNAs have been shown to: release paused Pol II by binding to and titrating away NELF (Schaukowitch et al. 2014), capture transcription factors (Sigova et al. 2015), and assist in chromatin loop formation (Li et al. 2013).

We are interested in the function of eRNAs during pioneer and lineage-specific transcription factor expression as transcription factors can determine differentiation and maintenance of cellular identity. CCAAT enhancer-binding protein alpha (CEBPA) is one of six C/EBP family members (CEBPα–CEBP*ζ*). The 42 and 30 kDa CEBPA isoforms are lineage-specific transcription factors that are also involved in metabolism (Wang et al. 1995) and cell cycle inhibition (Timchenko et al. 1996). Both isoforms play important roles in differentiation and cellular identity maintenance during and after hematopoiesis (Avellino et al. 2016), adipogenesis (Lefterova et al. 2008), and hepatogenesis (Westmacott et al. 2006). Because of its role in cell cycle inhibition and cellular differentiation, abnormal CEBPA function or regulation is often found in acute myeloid leukemia (AML), liver cancer, breast cancer, and lung cancers (Lourenco and Coffer 2017).

*CEBPA* regulation is well studied at the transcriptional level with regulatory roles described for key transcription factors (Avellino and Delwel 2017), upstream promoter methylation (Lu et al. 2010), a divergently transcribed long noncoding RNA (Xiao et al. 2015), and enhancer elements (Avellino et al. 2016). Avellino and colleagues noted that the *CEBPA* locus harbors 14 differentially active enhancers. Of particular interest to our study, the +9 kb enhancer appears to be utilized in a broad range of tissues and cell types. However, they did not investigate whether these enhancers were actively being transcribed and, if so, whether the resulting eRNAs are required for enhancer function.

Given the role of enhancer-mediated regulation of *CEBPA* (Avellino et al. 2016), we became interested in an elncRNA, (e_lncRNA|lncRNA_intergenic|CATG00000039093.1, FANTOM5 project, RIKEN) (Hon et al. 2017), that is transcribed from the sense strand of an enhancer located 9 kb downstream from the transcriptional start site (TSS) of human *CEBPA.* We have termed this elncRNA “CEBPA regulatory elncRNA downstream 9 kb” or “CRED9.” CRED9 detection, from cap analysis of gene expression-seq (CAGE-seq) data, only occurred in a subset of cell types tested including adipocytes and myeloid lineage cells and is positively correlated with CEBPA mRNA expression. (https://fantom.gsc.riken.jp/5/suppl/Hon_et_al_2016/vis/#/genes/CATG00000039093.1).

It is unknown whether CRED9 is required for +9 kb *CEBPA* enhancer activity. Therefore, the goal of this study was to determine if CRED9 has a role in regulating CEBPA expression. To accomplish this, reverse transcription droplet digital PCR (RT-ddPCR) was performed to establish a positive correlation between CRED9 and CEBPA mRNA expression in multiple cancer cell lines. Knockdown experiments showed that loss of CRED9 results in lower levels of CEBPA mRNA in Hep3B cells and CEBPA protein in both Hep3B and HepG2 cells. VPR fused to dCas9 (CRISPR activation) and directed upstream of CRED9 or *CEBPA* TSS confirmed that increased expression of either transcript correlated with an increase of the other in HeLa cells. Treatment with a short activating RNA (saRNA) that induces transcription of CEBPA mRNA also increased CRED9 expression in HepG2 and HeLa cells. This result suggests that the clinically relevant activity of CEBPA-saRNA is partially driven by increased CRED9 expression. Finally, supporting the findings in Bose et al. (2017), ChIP RT-qPCR of H3K27ac at the +9 kb *CEBPA* enhancer following knockdown of CRED9 in Hep3B cells revealed a 57.8% reduction in H3K27ac occupancy. Taken together, we assert that CRED9 transcript is required for maintaining a proper H3K27ac state at the +9 kb *CEBPA* enhancer and for proper expression levels of *CEBPA*.

## RESULTS

### Multiple downstream *CEBPA* enhancers are conserved among mammals and are actively transcribed

**FIGURE 1.**
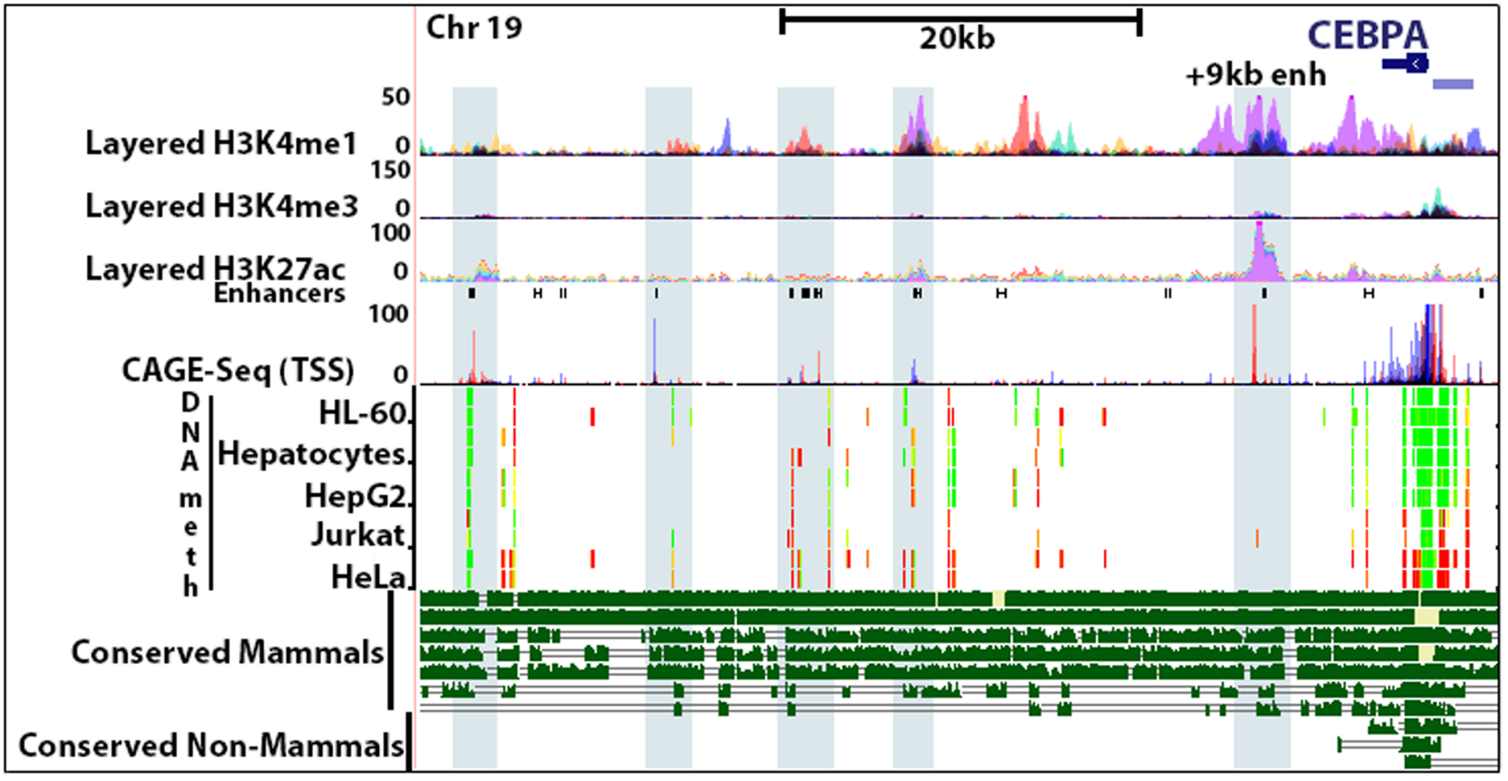
Enhancer predictive markers H3K4me, H3K4me3, and H3K27ac are depicted as an overlay of ChIP-seq data from GM12878, H1-hESC, HSMM, HUVEC, K562, NHEK, and NHLF cell lines (ENCODE). Predicted enhancers are demarcated as black rectangles (FANTOM5–RIKEN). CAGE-seq data (FANTOM5–RIKEN) shows sites of active transcription (red: positive strand, blue: negative strand). The +9 kb enhancer shows a high degree of active transcription initiation. Light blue rectangles highlight overlap between downstream *CEBPA* enhancers and sites of active transcription. DNA methylation (ENCODE) of the *CEBPA* promoter has previously been shown to suppress CEBPA expression (methylation status: green—low, yellow—intermediate, red—high). Some enhancers overlap with sites of CpG methylation while others, like the +9 kb, do not. Mammalian *CEBPA* enhancers (chimp, rhesus, mouse, dog, elephant, opossum, and platypus) show broad sequence conservation across all species, including the +9 kb enhancer (magenta rectangle). In contrast, nonmammals (chicken, lizard, zebrafish) lack the same enhancer sequences. (See Materials and Methods for track information.)

Our first objective was to identify transcribed enhancers of *CEBPA.* Prior work has identified multiple upstream and downstream enhancers of *CEBPA* (Avellino et al. 2016). These enhancers are categorized as either “cell-type specific,” for example, the myeloid specific +42 and +34 kb enhancers, or “general,” for example, the +9 kb enhancer. Identification of these enhancers was based on high levels of H3K27ac, a marker associated with euchromatin that overlaps with enhancer regions. To expand on this, we utilized publicly available ChIP-seq data (ENCODE) (Ernst et al. 2011; The ENCODE Project Consortium 2012; Wang et al. 2012; Davis et al. 2018) of H3K4me1, HeK4me3, H3K27ac, and CAGE-seq data to identify which enhancers show active transcription (Fig. 1). We noted that many of the enhancers have regions of conserved sequence between monotremes, marsupials, and placental mammals.

DNA methylation along the distal promoter (−645 bp to −1336 bp from the TSS) has been shown to be inversely correlated with CEBPA expression (Lu et al. 2010; Fasan et al. 2013). HepG2, primary hepatocytes, and HL-60 have lower levels of upstream promoter methylation and show moderate to high levels of CEBPA expression while Jurkat and HeLa cells are highly methylated and express low levels of CEBPA (Fig. 1). Methylation of enhancer DNA, like gene promoter methylation, can also inhibit transcription (Carrio et al. 2015; Liu et al. 2016). The +9 kb *CEBPA* enhancer is devoid of CpG methylation, while some downstream *CEBPA* enhancers contain multiple sites of differential methylation among different cell lines (Fig. 1). With these observations in consideration, the +9 kb *CEBPA* enhancer was flagged for further exploration due to being generally utilized, conserved, unmethylated, and transcribed.

### Transcriptional machinery occupancy of *CEBPA* enhancers differs between cell types

**FIGURE 2.**
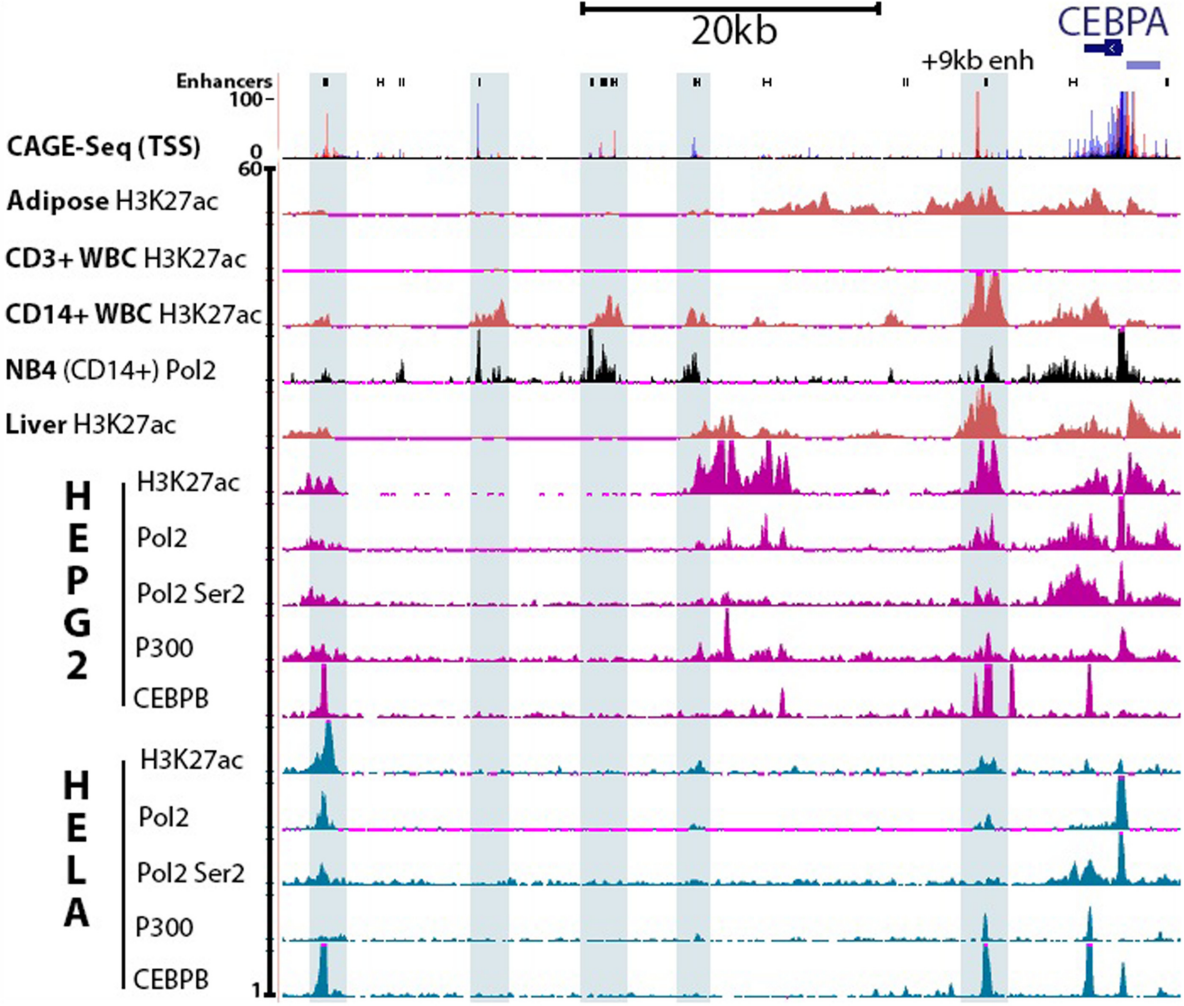
Analysis of “active” enhancers (H3K27ac) and active transcription (Pol II occupancy and CAGE-seq data) reveals differences between human cell types. Predicted enhancers are demarcated as black rectangles (FANTOM5–RIKEN). CAGE-seq data (FANTOM5–RIKEN) shows sites of active transcription (red: positive strand, blue: negative strand). CAGE-seq data is shown as an amalgamation of reads from different primary cell samples, including monocytes and adipocytes, which show active transcription from enhancers. H3K27ac status is depicted for primary adipose, CD3+ WBCs (lymphoid origin), CD14+ WBCs of myeloid origin, and hepatocytes (Roadmap Epigenomics Project), as well as the cancer cell lines HepG2 and HeLa. Pol II (ENCODE) and Pol II Ser2 signals overlap with CAGE-seq reads at enhancers (blue highlights). P300 binding is included as an additional marker of enhancers. CEBPB binding is one example of a transcription factor which binds to one or more of the *CEBPA* enhancers. A clear difference in H3K27ac, Pol II, and Pol II Ser2 status at the CRED9 is observed between HepG2 (CEBPA expressing) and HeLa (CEBPA suppressed). (See Materials and Methods for track information.)

CEBPA is expressed in adipose, myeloid-derived white blood cells (WBC), and hepatocytes. In contrast, CEBPA is down-regulated in lymphoid-derived WBCs. To determine if the occupancy of transcriptional machinery at *CEBPA* enhancers is also predictive of CEBPA expression we aligned CAGE-seq reads with H3K27ac signals (Bernstein et al. 2010) (ENCODE and Roadmap Epigenomics Project) from primary tissues including adipose, lymph origin CD3+ WBCs, myeloid origin CD14+ WBCs, and liver (Fig. 2). As predicted, cells that express CEBPA show high levels of H3K27ac signal while CD3+ WBCs do not. Additionally, all CAGE-seq peaks overlap with H3K27ac signals. To determine if transcriptional machinery overlaps with H3K27ac signal, we utilized a Pol II ChIP-seq track (ENCODE) of NB4, a CD14+ acute promyelocytic leukemia cell line, with the H3K27ac ChIP-seq tracks of CD14+ WBCs. Pol II signal from the myeloid derived NB4 cell line clearly overlaps with the H3K27ac signal of myeloid derived CD14+ WBCs, including at sites of myeloid specific enhancers +42 and +34 kb from the *CEBPA* TSS.

Next, we compared binding profiles of *CEBPA* enhancers between the CEBPA-expressing hepatocellular carcinoma (HCC) cell line HepG2 and non-CEBPA-expressing cell line HeLa by aligning ChIP-seq tracks (ENCODE) of H3K27ac, Pol II, Pol II Ser2, p300, and CEBPB. Focusing on the +9 kb CEBPA enhancer, H3K27ac is positively correlated with CEBPA expression: with HepG2 exhibiting a much stronger signal than HeLa. Similarly, both Pol II and a marker of polymerase 2 procession, Pol II Ser2, have stronger signals in HepG2 than in HeLa. p300, together with CBP, are acetyltransferases that acetylate H3K27 and are considered markers of enhancers (Visel et al. 2009). While p300 signal is higher in HepG2, HeLa also exhibits p300 binding at the +9 kb *CEBPA* enhancer. Lastly, we included CEBPB to represent one of many transcription factors that bind to the +9 kb enhancer. In both cell lines, CEBPB signal is observed at the +9 kb enhancer. Taken together, these observations led us to hypothesize that expression of enhancer RNA from the +9 kb *CEBPA* enhancer would positively correlate with expression of CEBPA mRNA.

### Identifying the 5′ transcriptional start site of CRED9 in Hep3B cells

**FIGURE 3.**
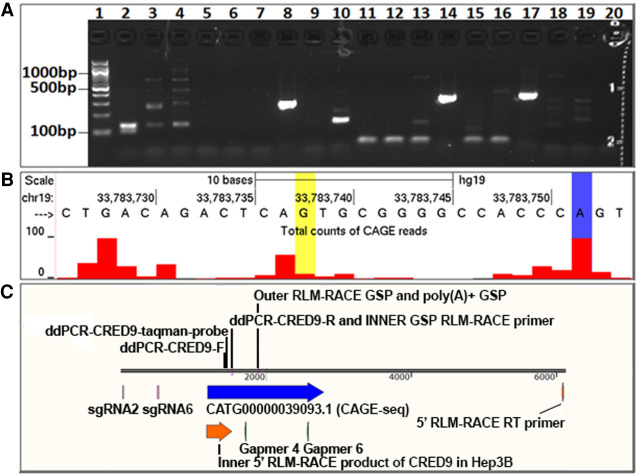
(*A*) The transcriptional start site of CRED9 was detected in Hep3B cells by performing 5′ RLM-RACE. CRED9 –specific PCR products from the second round of nested PCR in lanes 8, 14, and 17 were generated from the same reverse transcription product (same gene-specific RT primer). The successful RT primer annealed +5022 nt downstream from the TSS of CRED9. Other lanes include unsuccessful amplification of CRED9 5′ RACE products resulting from RT primers: random hexamers (lane *2*), oligo dT (lane *3*), NoRT (lane *20*), and gene-specific primers (all other lanes). (*B*) PCR amplified product from lanes *8, 14*, and *17* were cloned into sequencing plasmids. Sequencing and subsequent alignment of the products showed different transcriptional start sites between Hep3b (yellow highlight), annotated transcript CATG00000039093.1 (blue highlight), and CAGE-seq data (red peaks). (*C*) Schematic of the CRED9 and the +9 kb CEBPA enhancer genomic region. 5′ RLM-RACE product (red), CATG00000039093.1 CAGE-seq transcript (blue), single guide RNAs (magenta), CRED9-specific antisense oligonucleotide gapmers (green). Primers used for 5′ RLM-RACE, droplet digital PCR, and ChIP-qPCR are also depicted.

e_lncRNA|lncRNA_intergenic|CATG00000039093.1, a portion of CRED9 including a 5′ TSS, has previously been found in adipocytes and monocytes and is currently cataloged in the FANTOM5 database (RIKEN) (Hon et al. 2017). Given the binding and histone profile of +9 kb CEBPA enhancer in hepatocytes and HepG2 we reasoned that CRED9 should be detectable in the HCC cell line Hep3B. We first performed RNA ligase-mediated rapid amplification of cDNA ends (RLM-RACE) on 10 µg of total RNA to determine if CRED9 is expressed in Hep3B and from where it originates (Fig. 3A).

**FIGURE 4.**
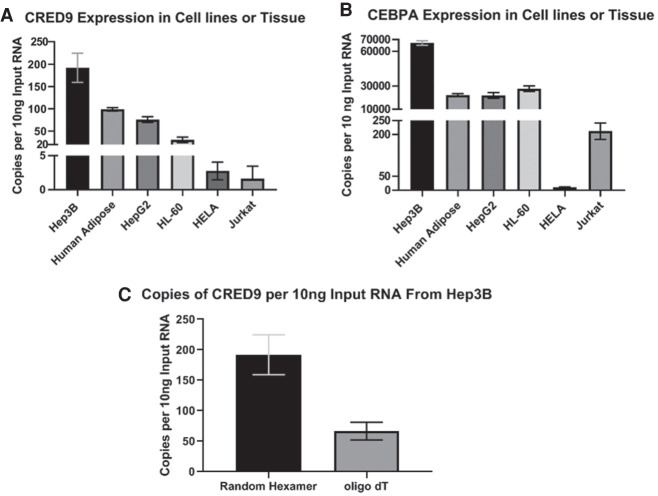
RT-ddPCR reveals a direct correlation between the expression level of (*A*) CRED9 and (*B*) CEBPA mRNA in Hep3B, Human Adipose, Hepg2, HL-60 (myeloid origin), HeLa, and Jurkat (lymphoid origin). (*C*) Comparison of CRED9 copy number from cDNA generated by random hexamers or oligo d(T) RT primers. (*n* = 3 biological replicates for all cell lines; *n* = 2 technical replicates of a 25-person pool of adipose RNA; normalized to GAPDH mRNA; error bars: std).

Disappointingly, attempts using multiple gene-specific primers as well as random hexamers and oligo (dT) were not successful (Fig. 3A). This is likely due to target transcript 5′ or general instability paired with the inefficiencies of the 5′ RLM-RACE protocol which requires decapping of m7G and subsequent ligation of a unique 5′RACE adaptor oligonucleotide sequence to freshly decapped RNA. The gene-specific reverse transcription primer (Fig. 3C) that was successful at producing product from two subsequent nested PCRs annealed 5022 nt downstream from the TSS that we identified by RLM-RACE (Fig. 4B). The ability to produce this product was a fortuitous event given the lack of success with the other reverse primers. However, we are confident in its validity as cloning into pCR-Blunt using the Zero Blunt PCR Cloning Kit #K270020 (Thermo Fisher) and the subsequent sequencing of the cloned 5′ RLM-RACE product contains the unique adaptor sequence followed by sequence that perfectly aligns to the +9 kb CEBPA enhancer (sequencing data in Supplemental Material). Interestingly, the CRED9 TSS found in Hep3B begins 14 nt upstream of the currently annotated TSS for e_lncRNA|CATG00000039093.1 (Fig. 3B).

Attempts at determining the 3′ transcriptional end site of CRED9 in Hep3B by 3′ RACE were ultimately unsuccessful (data not shown), again possibly due to the low abundance of CRED9 and rapid turnover of eRNAs. Nevertheless, at least some CRED9 transcripts are transcribed >4000 nt long as determined by 5′ RLM-RACE, polyadenylated (Fig. 4C), and transcribed only from the +strand according to CAGE-seq data from multiple types of monocytes and adipocytes (Hon et al. 2017). Because of this, we are inclined to agree with the FANTOM5 consortium that e_lncRNA|CATG00000039093.1 is correctly labeled an elncRNA. Given that CRED9 is a modestly more defined form of the same transcript we believe it is also likely an elncRNA.

### CRED9 expression is directly correlated with CEBPA mRNA expression across multiple cell lines

eRNAs can be difficult to study as they are typically expressed in a cell-specific context and are often found at very low copy numbers even though their rates of transcription mirror those of other lncRNAs and mRNAs (Lam et al. 2014). The low copy number of eRNAs is attributed to their innate instability and rapid degradation by the nuclear exosome and the nonsense-mediated decay pathway (Arnold et al. 2020). Counter-intuitively, polyadenylation of eRNAs can lead to greater instability due to exosome recruitment, RNAP II instability, and transcription termination (Henriques et al. 2018). Low steady-state copy number of a target causes two major, though noninsurmountable, issues in routine qPCR detection. First, residual PCR inhibitors from the prior reverse transcription step, which cannot be diluted out, can inhibit PCR efficiency. Second, late quantification cycles of Cq ≥ 29 are unavoidable, and therefore very efficient cDNA amplification is required in order to maintain a linear dynamic range at later cycles. Indeed, we were unable to acquire satisfactory results when we attempted to detect steady-state CRED9 from Hep3B cells using TaqMan-based qPCR. To overcome this limitation, we opted to use droplet digital PCR (ddPCR). PCR efficiency and low target copy numbers are not major limitations when performing ddPCR since target amplification is run to endpoint prior to detection.

To detect CRED9, we developed a custom TaqMan-based ddPCR assay (Fig. 3C) (Materials and Methods). We paired this assay with predesigned CEBPA and GAPDH ddPCR assays (Bio-Rad) in order to compare CRED9 expression to CEBPA mRNA expression in RNA isolated from pooled human adipose tissue (Takara Bio), Hep3B, HepG2, HL-60, HeLa, and Jurkat cells. GAPDH was used for normalization and allowed us to convert copies of target per well to a readout of copies per nanogram of input RNA (Coulter 2018). We set RNA input to 10ng for ease of graphical representation. Steady states of CRED9 across all samples are sub copy per cell, likely due to the inherent instability of eRNAs. Still, taken in the context of a population of cells we found that steady-state CRED9 expression is quantifiable and positively correlated with CEBPA mRNA expression in adipose and all cell lines tested (Fig. 4A,B). Additionally, RT-qPCR using oligo(dT) as a reverse primer revealed that at least some fully transcribed CRED9 transcripts are polyadenylated in Hep3B (Fig. 4C), though, not as readily detectable as products produced by random hexamers.

Admittedly, we cannot fully rule out that some of the random hexamer or oligo (dT)-derived transcripts arise from other transcripts that run either sense or antisense through the same region. This experimental caveat is rather common when attempting to amplify a specific target as multiple sense or antisense transcripts are transcribed through the same genomic region and will ultimately be amplified during PCR if present. However, CAGE-seq (FANTOM5), RLM-RACE data (Fig. 3A), and ASO-mediated CRED9-specific knockdown (discussed next) argue that we are most likely making observations regarding CRED9.

### Knockdown of CRED9 in Hep3B causes a reduction in CEBPA and albumin mRNA expression and an increase in CEBPG mRNA expression

After establishing a positive correlation between CRED9 and CEBPA mRNA expression, we next asked whether CRED9 has biological function or is merely a nonfunctional product of Pol II-accessible DNA. We chose Hep3B cells because they expressed the highest level of steady-state CRED9 and CEBPA mRNA in the cell lines we tested and are relatively easy to transfect. To test functionality, we sought antisense oligonucleotide gapmers that were capable of selectively knocking down CRED9 expression. We initially screened six ASO gapmers for their ability to knock down CRED9. Of the six ASO screened, we selected gapmer 4 (Gap4) and gapmer 6 (Gap6) which hybridize to CRED9 starting at 428 and 1387 nt, respectively, downstream from the 5′ end of the CRED9 transcript (Fig. 3C).

**FIGURE 5.**
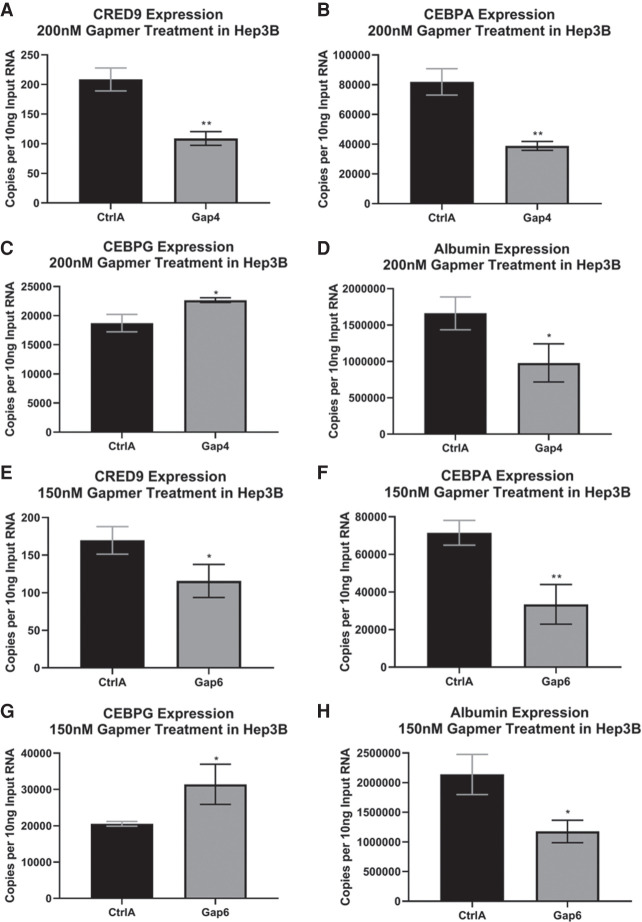
Knockdown of CRED9 results in lower CEBPA and albumin mRNA expression as well as an increase in CEBPG expression in Hep3B cells as measured by RT-ddPCR (*A*,*E*). Knockdown of CRED9 using 200 nM gapmer 4 or 150 nM gapmer 6 and the resulting changes in (*B*,*F*) CEBPA, (*C*,*G*) CEBPG, and (*D*,*H*) albumin mRNA expression. (*n* = 3 biological replicates; normalized to GAPDH mRNA; error bars: std; *P*-values from unpaired *t*-tests where significance “*” cut-off is [*] *P* ≤ 0.05, [**] *P* ≤ 0.01, [ns] not significant).

Knockdown of CRED9 in Hep3B cells was achieved by reverse transfection using 200 nM final concentration of Gap4 or 150 nM of Gap6. A nontargeting ASO “CtrlA” (Qiagen) transfected at either 150 or 200 nM served as an ASO treatment control. Cells were harvested after 24 h of incubation and measured for changes in transcript abundance by RT-ddPCR. Final gapmer concentration was limited based on maximal CRED9 knockdown versus unacceptable changes to reference gene (GAPDH). We found that gapmer 4 treatment resulted in a fold change of −1.91 (CRED9) and −2.11 (CEBPA mRNA), relative to control (Fig. 5A,E). Gapmer 6 treatment resulted in a fold change of −1.47 (CRED9) and −2.14 (CEBPA mRNA), relative to control (Fig. 5B,F). This result shows that CRED9 has a positive regulatory role on CEBPA mRNA expression and knockdown of this eRNA results in less CEBPA mRNA expression.

Direct knockdown of CEBPA in Hep3B cells can inhibit cell proliferation beyond 24 h post transfection with anti-CEBPA shRNA plasmids (Lu et al. 2010). We did not, however, observe a reduction in proliferation in Hep3B cells after CRED9 knockdown beyond a very modest lipofection-specific proliferation inhibition. We also did not see significant changes in our calibrator gene GAPDH. This is likely owing to limiting our time point to only 24 h and the indirect knockdown of CEBPA taking longer than directly targeting CEBPA mRNA with ASO gapmers.

In addition to CEBPA mRNA, we looked at mRNA expression levels of two genes which are either negatively or positively regulated by CEBPA protein activity. Previous work has shown that the +9 kb *CEBPA* enhancer can make physical contact with the *CEBPG* promoter (Avellino et al. 2016). Using cDNA from the same samples, we used ddPCR to detected changes in CEBPG expression and found a fold change of 1.21 using gapmer 4 and 1.53 using gapmer 6, relative to control (Fig. 5C,G). Because CEBPA can suppress CEBPG expression, we attributed the increase in CEBPG mRNA to a likely decrease in CEBPA protein (Alberich-Jorda et al. 2012). We also concluded that CRED9 does not positively regulate CEBPG in Hep3B cells because we did not detect a decrease in CEBPG expression following CRED9 knockdown.

We chose albumin as an example gene that is positively regulated by CEBPA because it is highly expressed in Hep3B and is a well-known target of CEBPA transactivation. We observed a fold change in albumin mRNA of −1.70 using gapmer 4 and −1.82 using gapmer 6, relative to control (Fig. 5D,H).

Surprisingly, while gapmer 6 was less effective at knocking down steady-state CRED9, we observed larger changes in CEBPA, CEBPG, and albumin expression. One potential explanation for this is that, while not being as efficient at knocking down steady state CRED9, gapmer 6 might be more potent at targeting and reducing the functional pool, likely a nascently transcribed transcript, of CRED9. Another potential explanation is that hybridization of gapmer 6 to CRED9 disrupts a functional domain, if such a domain exists, within the CRED9 structure without necessarily causing RNase-H-mediated cleavage. Regardless, these knockdown results demonstrate the causal relationship of CRED9 as a positive regulator of CEBPA expression.

### ASO-mediated knockdown of CRED9 in Hep3B does not alter expression of genes that are not directly or predicted to be indirectly regulated by CEBPA

Changes in the concentration of a lineage-specific transcription factor, such as CEBPA, can have transcriptome-wide consequences which complicate interpretations involving the relationship between enhancer-mediated and transcription factor-mediated regulation events. Genes located ±320 kb of *CEBPA* have known CEBP family member binding sites in or near their promoters. Some genes are simply not expressed at appreciable levels in liver or HCC cell lines.

**FIGURE 6.**
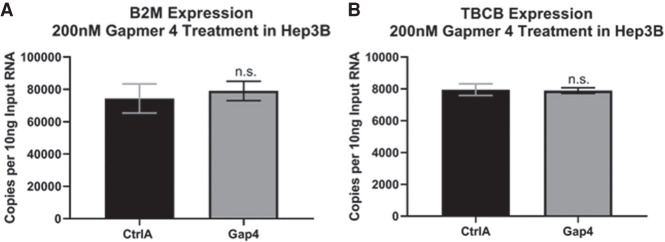
Knockdown of CRED9 using 200 nM of gapmer 4 does not cause a change in expression of B2M (Chr: 15) or TBCB (Chr: 19) mRNA as measured by RT-ddPCR (*A,B*). (*n* = 3 biological replicates; normalized to GAPDH mRNA; error bars: std; *P*-values from unpaired *t*-tests: [ns] not significant).

Instead of determining which genes near the +9 kb *CEBPA* enhancer remain unaffected by CRED9 knockdown, something RNA-seq would be more efficient at, we sought to rule out possible nonspecific effects of ASO treatment by performing RT-ddPCR using primer probe assays (Bio-Rad, Inc.) for B2M (located on Chr: 15) and TBCB (located on Chr: 19, 2.8 mb from *CEBPA*). Both of these genes were chosen due to previously being described as “housekeeping genes” and because subsequent analysis of publicly available ChIP-seq data showed that their promoters had minimal potential to be bound by CEBP family members. They are also sufficiently far away from the +9 kb *CEBPA* enhancer and therefore unlikely to be affected by it. As predicted, knockdown of CRED9 by 200 nM of gapmer 4 did not cause a significant change in B2M or TBCB mRNA expression (Fig. 6A,B).

### Knockdown of CRED9 leads to a reduction in CEBPA protein in Hep3B and HepG2 cells

**FIGURE 7.**
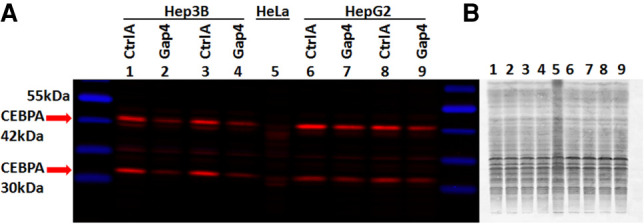
Knockdown of CRED9 by 200 nM ASO gapmer 4 causes a reduction in CEBPA protein expression. Detection by fluorescent western blot: (*A*) Lanes, 1: CtrlA, 2: Gap4, 3: CtrlA, 4: Gap4, 5: HeLa lysate, 6: CtrlA, 7: Gap4, 8: CtrlA, 9: Gap4. *Upper* red arrow: CEBPA 42 kDa isoform. *Lower* red arrow: CEBPA 30 kDa isoform. Hep3B: 42 kDa (FC: −2.20, CV: 6.33%) 30 kDa (FC: −3.18, CV: 6.21%). HepG2: 42 kDa (FC: −1.52, CV: 14.44%) 30 kDa (FC: −1.43, CV: 30.12%). Reduced expression of CEBPA in Hep3B is robust for both isoforms. Knockdown in HepG2 is more subtle, likely due to less efficient transfection. (*B*) Revert 700 total protein stain of the membrane from *A* used for total protein normalization. (*n* = 2 biological replicates, [FC] fold change, [CV] coefficient of variation).

Decreased CEBPA mRNA expression appears to cause changes in downstream gene expression as evidenced by the changes in CEBPG and albumin mRNA following CRED9 knockdown (Fig. 5). To ensure these changes are the result of CEBPA protein reduction we performed a fluorescent western blot assay using protein isolated from Hep3B and HepG2 cells. Cells were reverse transfected with 200 nM final concentration of either control gapmer or gapmer 4 for 24 h (Fig. 7A). Prior to the blocking step, we stained the same membrane with Revert 700 total protein stain (Li-cor) and acquired a near IR image for the purpose of protein loading and transfer normalization (Fig. 7B). We observed a −2.20-fold change of the 42 kDa CEBPA isoform and a −3.18-fold change of the 30 kDa CEBPA isoform in protein isolated from Hep3B cells treated with gapmer 4, relative to control. In HepG2, we observed a −1.52-fold change of 42 kDa CEBPA. We also observed a −1.43-fold change of 30 kDa CEBPA in HepG2 but the coefficient of variation was too high for the observation to be conclusive. In our hands, HepG2 has been difficult to transfect which might contribute to the weaker knockdown observed. Nevertheless, the decrease in CEBPA protein following CRED9 knockdown shows that CRED9 also influences CEBPA expression in HepG2. Taken together, we conclude that knockdown of CRED9 causes a reduction of CEBPA protein expression.

### Increasing the expression level of either CRED9 or CEBPA mRNA correlates with an increase in the expression of the other

We wanted to determine if increased expression of either CRED9 or CEBPA mRNA influenced the expression of the other transcript. To accomplish this, we cotransfected HeLa cells with a pair of plasmids expressing single guide RNAs (sgRNAs) that target either: upstream of the CRED9 TSS, upstream of the *CEBPA* TSS, or control sgRNAs targeting luciferase (nonexistent in mammals) and part of the mitochondrial genome (inaccessible compartment). Each pair of sgRNA plasmids was cotransfected along with a plasmid that expresses dCas9-VPR, a fusion protein that lacks Cas9 cleavage activity but can be directed by guide RNA to induce site-specific transcriptional activation through a fused VPR domain (Chavez et al. 2015). We chose HeLa cells to transfect into because they exhibit very low CRED9 expression and have minimal CEBPA mRNA expression.

**FIGURE 8.**
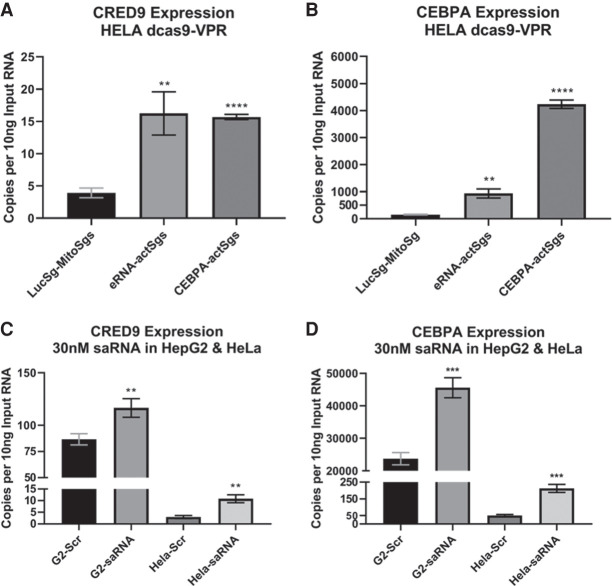
(*A*) CRED9 and (*B*) CEBPA mRNA expression in HeLa cells following cotransfection of dCas9-VPR expression plasmid and either sgRNA expression plasmids targeting upstream of CRED9 TSS or the promoter of *CEBPA*. (*C*) CRED9 and (*D*) CEBPA mRNA expression in HepG2 or HeLa cells following transfection of 30 nM CEBPA short activating RNA. In both experiments, increased expression of one transcript correlated with increased expression of the other. (*n* = 3 biological replicates; normalized to GAPDH mRNA; error bars: std; *P*-values from unpaired *t*-tests where significance “*” cut-off is *P* ≤ 0.05, [**] *P* ≤ 0.01, [***] *P* ≤ 0.001, [****] *P* ≤ 0.0001, [ns] not significant).

**FIGURE 9.**
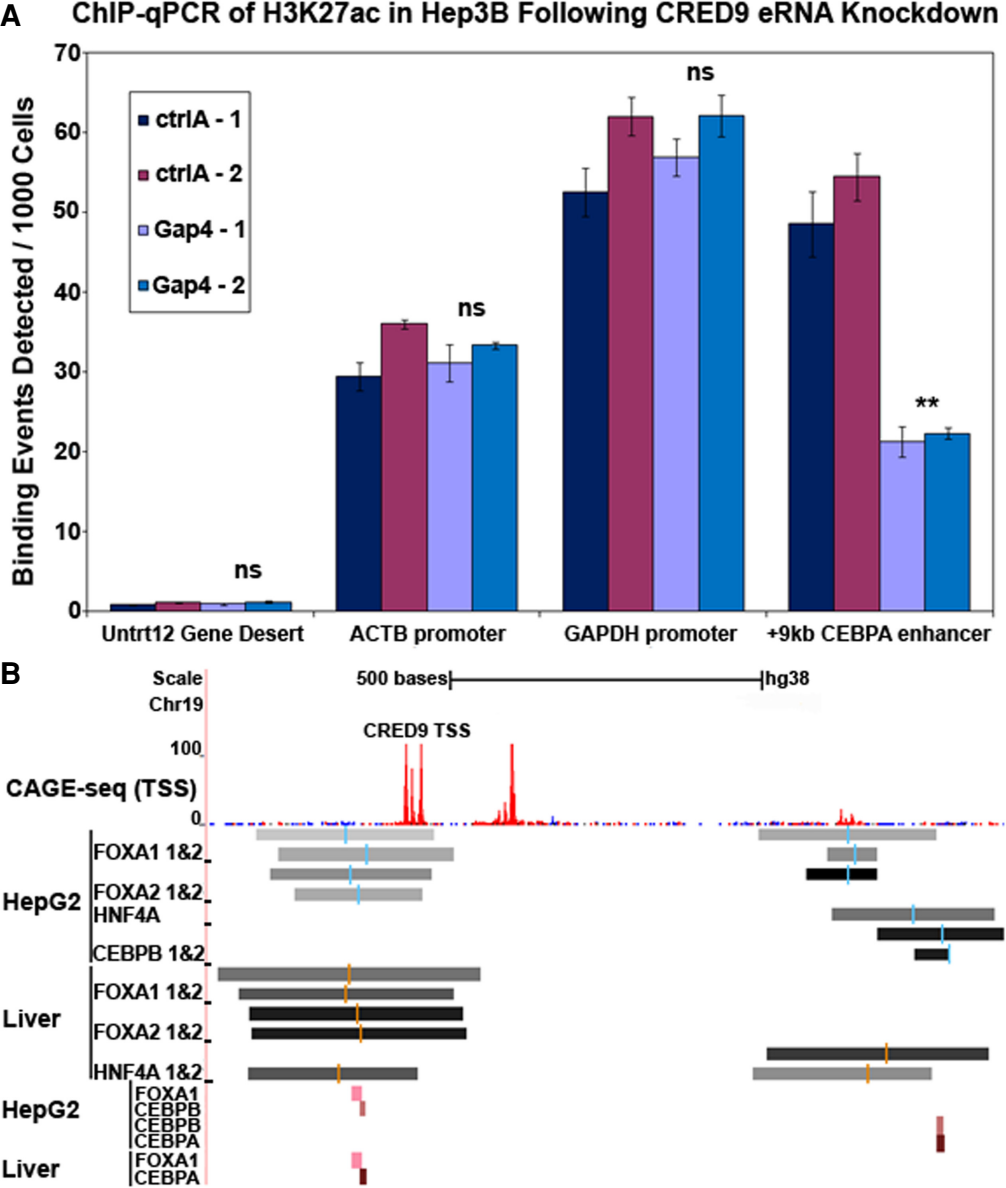
(*A*) CRED9 knockdown in Hep3B cells with 200 nM gapmer 4 reveals a loss of H3K27ac at the +9 kb *CEBPA* enhancer as measured by ChIP-seq RT-qPCR. Control H3K27ac-enriched regions within the *ACTB* and *GAPDH* promoters do not change upon CRED9 knockdown. Minimal amplification of a “gene desert” product within Chr: 12, devoid of H3K27ac, confirmed low nonspecific pull-down with the H3K27ac-specific antibody. (*B*) ChIP-seq binding profiles of liver-enriched transcription factors HNF4A, FOXA1, FOXA2, CEBPA, and CEBPB are depicted around the TSS of CRED9. The binding profiles clearly demonstrate that liver-enriched transcription factors bind the +9 kb *CEBPA* enhancer in liver and a hepatocellular carcinoma cell line. (CAGE-seq data [Fantom5 project, RIKEN]), ChIP-seq data: Degree of shading is indicative of the degree of transcription factor binding from lower (lighter) to higher (darker). Blue and red bars indicate transcription factor consensus sequence (ENCODE). Smaller rectangles represent binding sites of the indicated transcription factors from a separate data source (Unibind). (*n* = 2 biological replicates; error bars: std; *P*-values from unpaired *t*-tests where significance “*” cut-off is [**] *P* ≤ 0.01, [ns] not significant).

We initially thought that inducing expression of CRED9 would lead to an increase of CEBPA mRNA expression whereas inducing CEBPA mRNA production would not result in a change of CRED9 expression. To our surprise, we found that inducing the expression of either target led to a similar fold change of steady-state CRED9 of ∼4.00, relative to control (Fig. 8A). However, when observing the effect on CEBPA mRNA expression we found that targeting the +9 kb *CEBPA* enhancer led to a 6.09-fold change in CEBPA expression while directly targeting the *CEBPA* promoter led to a 27.59-fold change in CEBPA expression, relative to control (Fig. 8B). Due to PAM sequence limitations, the CRED9 sgRNAs bind further upstream of the CRED9 TSS than the *CEBPA* sgRNAs relative to the *CEBPA* TSS. Thus, we anticipated this discrepancy to result in weaker transcriptional activation of CRED9 versus CEBPA mRNA (Boettcher and McManus 2015). Given the 9 kb distance between the *CEBPA* promoter and +9 kb *CEBPA* enhancer, the dCas9-VPR constructs might be able to directly influence both sites simultaneously. Alternatively, increased CEBPA protein expression may influence the expression of CRED9 since CEBP family members form heterodimers and CEBPB binds to the +9 kb *CEBPA* enhancer (Figs. 2, 9). In support of this, a recent study found that CEBPB protein induces the expression of a *CEBPB* eRNA and the same eRNA positively regulates *CEBPB* expression (Xiong et al. 2019). Additionally, we noted CEBPA binding sites within the +9 kb *CEBPA* enhancer (Fig. 9B). Paired with the results from knockdown experiments, we believe it is probable that increased expression of one transcript influences the expression of the other. It would be informative to perform both CEBPA overexpression and knockdown experiments to determine the role of CEBPA protein in the regulation of CRED9.

As an alternative method, we utilized a short activating RNA (saRNA) that has previously been shown to induce CEBPA expression (Voutila et al. 2017). CEBPA saRNA is also currently under investigation in the clinic for the treatment of HCC (Sarker et al. 2020). saRNAs differ from siRNAs by binding to complementary chromatin-bound RNA, and possibly DNA, near or within a target gene's promoter but do not necessarily induce target RNA cleavage. Rather, through an Argonaut 2 (AGO2)-specific process, the resulting saRNA–AGO2 complexes induce transcriptional gene activation. The CEBPA-saRNA utilized here is thought to bind in an AGO2-dependent manner to an antisense transcript that is transcribed through the *CEBPA* coding region (DNA homology site is −1099 bp the CEBPA TSS) (Voutila et al. 2017). Here, 30 nM final concentration of CEBPA-saRNA or a scrambled sequence control were reverse-transfected into HepG2 or HeLa cells for 48 h prior to RNA isolation. We observed fold changes of 1.35 (HepG2) and 3.71 (HeLa) expression in CRED9, relative to scrambled control (Fig. 8C). We also observed fold changes of 1.92 (HepG2) and 4.26 (HelA) expression in CEBPA mRNA, relative to scrambled control (Fig. 8D). The changes in expression for both transcripts increased in HepG2 and HeLa showing a positive correlation between increased CEBPA mRNA expression and CRED9 expression following saRNA treatment.

It would be mechanistically interesting in the future to determine if CEBPA-saRNA directly influences the +9 kb *CEBPA* enhancer which in turn influences the CEBPA promoter, or if saRNA-induced increase in CEBPA protein promotes CEBPA binding to the +9 kb *CEBPA* enhancer which in turn increases the expression of CRED9.

### Knockdown of CRED9 in Hep3B causes a reduction of H3K27ac at the +9 kb *CEBPA* enhancer

Nascently transcribed eRNAs have been demonstrated to stimulate HAT activity of CBP/p300 by binding to an RNA-binding region in the HAT domain of CBP which, in turn, leads to higher levels of H3K27ac at the associated enhancer (Bose et al. 2017). To test if CRED9 is required for maintaining H3K27ac at the +9 kb *CEBPA* enhancer, we knocked down CRED9 in Hep3B cells via transfection with 200 nM of gapmer 4 for 24 h. After 24 h cells were fixed and prepared for chromatin IP (Materials and Methods). Frozen cell pellets were sent to Active Motif, HistonePath ChIP-qPCR service (cat# 25014) to be further processed and analyzed for changes in H3K27ac status at the +9 kb *CEBPA* enhancer (Materials and Methods).

ChIP RT-qPCR of H3K27ac at the +9 kb *CEBPA* enhancer following knockdown of CRED9 revealed a 57.8% reduction in H3K27ac occupancy (Fig. 9A). Meanwhile, no significant changes in H3K27ac occupancy occurred at the positive control regions of the *ACTB* and *GAPDH* promoters (Fig. 9A). From this experiment we concluded that CRED9 is required for maintaining a high level of H3K27ac occupancy at the +9 kb *CEBPA* enhancer and reduction of H3K27ac occupancy following CRED9 knockdown results in lower enhancer activity.

### Differences in CRED9 expression among different cell types are likely a result of differential transcription factor binding profiles of the +9 kb *CEBPA* enhancer

p300 binds within the +9 kb *CEBPA* enhancer in both HepG2 and HeLa cell lines (Fig. 2). Despite this, H3K27ac, Pol II, and Pol II Ser2 signals are much higher at the +9 kb *CEBPA* enhancer in HepG2 than in HeLa cells (Fig. 2). CRED9 expression is also much higher in HepG2 than in HeLa cells (Fig. 4). To account for these differences, we looked at what liver-enriched transcription factors may occupy the +9 kb *CEBPA* enhancer in liver and liver-derived cell lines that would not be expressed in HeLa. Using publicly available ChIP-seq data from ENCODE and Unibind (Gheorghe et al. 2019), we found that FOXA1, FOXA2, HNF4A, CEBPB, and CEBPA bind either upstream of the CRED9 TSS or within the +9 kb *CEBPA* enhancer (Fig. 9B). Barring CEBPB (Fig. 2), we were unable to perform the same analysis for HeLa cells due to a lack of available ChIP-seq data for these transcription factors.

Instead, we compared RNA-seq expression results of these liver-enriched transcription factors between liver, HepG2, Hep3B, and HeLa cells using data obtained from protein atlas (Uhlen et al. 2017) and expression atlas (Petryszak et al. 2014). We found that these transcription factors, with the exception of CEBPB, are expressed at moderate-to-high levels in liver, HepG2, and Hep3B but are low or barely detected in HeLa (Table 1). We speculate that the differences in CRED9 expression level as well as the differences in Pol II occupancy, H3K27ac, and Pol II Ser2 signals between HepG2 and HeLa are due to these differences in transcription factor binding at the +9 kb *CEBPA* enhancer. However, to generalize this notion, further investigation will be needed to determine what transcription factors bind the +9 kb *CEBPA* enhancer in other cell types, for example, adipose and myeloid lineage cells, which also express CRED9. Alternatively, currently unknown inhibitory factors might be present at the +9 kb *CEBPA* enhancer in cells that do not express CRED9.

**TABLE 1.**
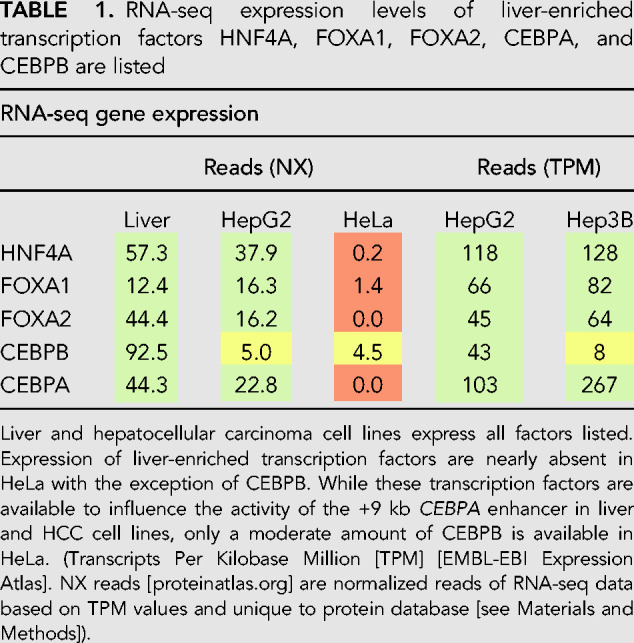
RNA-seq expression levels of liver-enriched transcription factors HNF4A, FOXA1, FOXA2, CEBPA, and CEBPB are listed

## DISCUSSION

eRNAs number in the tens of thousands (Hon et al. 2017). Although eRNAs can be identified using RNA-seq methods, determining the function of a particular eRNA requires target-specific testing. To date, only a small fraction of eRNAs have been studied and shown to have a role in gene regulation (Arnold et al. 2020). Many studies that have determined regulatory functions for eRNAs remain unresolved mechanistically (Arnold et al. 2020). The difficulty in assigning a mechanism to eRNAs is that no single explanation for their function seems sufficient. Indeed, various mechanisms have been described for different eRNAs and it is possible that any combination of the currently known mechanisms or ones yet to be discovered contribute to the function of a given eRNA (Sigova et al. 2015; The ENCODE Project Consortium et al. 2016; Bose et al. 2017; Le Noir et al. 2017; Arnold et al. 2020).

We were particularly intrigued by the model put forth by Bose and colleagues, whereby eRNAs are required for maintaining high H3K27ac levels through an RNA–CBP binding interaction that stimulates CBP HAT activity. To us, the generalized nature of this model places less importance on fully defining the sequence of a given eRNA transcript and, instead, emphasizes whether or not the presence of the eRNA transcript is required for enhancer activity. Extrapolating from this model, high levels of steady-state CRED9 would not be required for maintaining H3K27ac at the +9 kb *CEBPA* enhancer. Instead, transient expression of CRED9 would be sufficient to stimulate CBP HAT activity. In support of this, we found that CRED9 is necessary for maintaining higher levels of H3K27ac at the +9 kb *CEBPA* enhancer which explains how CRED9 regulates *CEBPA*.

Confoundingly, p300/CBP activity is required for eRNA transcription to occur in the first place (Raisner et al. 2018). A basal level of p300/CBP HAT activity may be necessary to prime enhancers for transcription but is not sufficient to drive enough eRNA transcription toward a stimulatory feedback loop. This may occur in HeLa cells at the +9 kb *CEBPA* enhancer where p300/CBP is bound but CRED9 is not expressed at a sufficient level (Figs. 2, 4A). Instead, the trigger for a higher rate of eRNA transcription from a poised enhancer likely requires binding of cell type-enriched transcription factors. This may occur in HepG2 cells at the +9 kb *CEBPA* enhancer (Figs. 2, 4A) where p300/CBP is bound and CRED9 is expressed at a sufficient level. Indeed, knockdown of FOXA1 can inhibit both eRNA production and enhancer activity where FOXA1 binds while leaving p300/CBP binding largely unchanged (Wang et al. 2011). In HepG2 cells, FOXA1, along with other lineage-specific transcription factors, binds to the +9 kb *CEBPA* enhancer (Fig. 9B), while in HeLa cells, the same set of transcription factors are unlikely to be bound due to low expression (Table 1).

Enhancer utilization is largely tissue specific. Consequently, eRNA expression is also largely tissue specific. In this respect, the +9 kb *CEBPA* enhancer is interesting as it is utilized in many, or possibly all, cell lines and tissues that express CEBPA at an appreciable level (Avellino et al. 2016). In this study, we show that CRED9 positively regulates CEBPA expression and that expression of CRED9 differs among cancer cell lines. In a broader context, genome-wide association studies have demonstrated that most disease-associated mutations occur outside of the coding genome. A majority of these mutations occur in enhancers and, in many cases, in associated eRNA transcripts (Farh et al. 2015; Aune et al., 2017). As a result, the field of medicine is showing a growing interest in disease-associated eRNAs. Efforts are being made to use eRNA expression profiles to flag and identify disease-specific variants (Arnold et al. 2020). While not explored in this study, we predict that a portion of patient tumors that exhibit suppressed CEBPA expression will also harbor *CEBPA* enhancer mutations and suppressed CRED9 expression. Ultimately, it will be critical to hasten the rate of functional eRNA identification in order to realize the diagnostic potential of transcriptome-wide eRNA profiling.

## MATERIALS AND METHODS

### Cell lines

HepG2 (ATCC HB-8065), Hep3B (ATCC HB-8064), HeLa (ATCC CLL-2), HL-60 (ATCC CLL-240), and Jurkat E6-1 (Aids reagent cat# 177) were purchased from the respective suppliers.

### Tissue culture

All cells were grown in RPMI 1640 without l-glutamine (Corning Cellgro) supplemented with a final concentration of 2 mM l-glutamine and 10% FBS. Adherent cells were passaged at ∼85% confluence by washing once with DPBS 1× (Corning) followed by incubation with Trypsin EDTA 1× solution (Irvine Scientific). Dilution of split cells was done according to ATCC recommendations. Suspension cells were maintained between 1 × 10^5^ and 1 × 10^6^ cells/mL and split into new flasks according to ATCC recommendations.

### Cell counting

Cells were counted on a Cellometer K2 (Nexcelom Bioscience) with the aid of Viastain AO/PI reagent (Nexcelom Bioscience #CS2-0105). Cell viability for all experiments was ≥90% prior to seeding.

### Custom oligonucleotide synthesis

All custom oligonucleotides were synthesized by Integrated DNA Technologies, Inc. (IDT) unless otherwise stated.

### RLM-RACE for 5′ transcriptional start site detection

FirstChoice RLM-RACE Kit (Thermo Fisher Scientific) was used according to the manufacturer's protocol. Additionally, 1 µL Super RNase inhibitor was added (1 µL to 19 µL CIP reaction). An amount of 10 µg of Hep3B total RNA was used. Various eRNA specific primers, random hexamer, or oligo d(T) were used as reverse primers. Primers: CRED9-specific Reverse Transcription Primer: 5′**-CTTCAAGGAAAGGGCAAGGGTAG**-3′; 5′ RACE Adapter Sequence: 5′-GCUGAUGGCGAUGAAUGAACACUGCGUUUGCUGGCUUUGAUGAAA-3′; Outer Adapter Sequence Primer: 5′-GCTGATGGCGATGAATGAACACTG-3′; Outer eRNA specific primer 3: 5′-**TAGCCTCCTGGAGCGATTTA**-3′; Inner Adapter Sequence Primer: 5′-CGCGGATCCGAACACTGCGTTTGCTGGCTTTGATG-3′; Inner eRNA Specific Reverse Primer 3: 5′-**TCTGAAGGCACTGTGGTCTG**-3′. PCR was performed using Phusion High-Fidelity Polymerase (NEB) according to manufacturer's protocol. PCR products were excised from gel and purified using a NucleoSpin Gel and PCR Clean-Up kit (Machery-Nagel). Cloning was performed using Zero Blunt PCR Cloning Kit #K270020 (Thermo Fisher). Plasmids from selected colonies were amplified in DH10b *E. coli* and purified by NucleoSpin Plasmid Mini kit for plasmid DNA 740588.50 (Machery-Nagel). Sequencing was performed by Eurofins Scientific. Alignment of sequenced clones was performed using Snapgene software (GSL Biotech LLC).

### RNA isolation

An amount of 300 µL of TRIzol (Thermo Fisher) was used per well for 24-well plates and 500 µL for six-well plates. RNA was isolated from TRIzol using the Direct-zol RNA MicroPrep kit (Zymo Research #R2060) according to the manufacturers’ protocol with the modification of using 21 µL RNase-free H2O during RNA elution. The protocol includes an on-column DNase treatment.

### Pooled human adipose RNA

Human adipose tissue total RNA 10 µg #636558 was purchased from Takara Bio Inc. Normal human adipose tissue pooled from 18 male/female Caucasians, ages: 22–61; cause of death: sudden death. Additionally, this RNA was treated with Turbo DNA-Free kit DNase (Thermo Fisher AM1907).

### DNase treatment

A second DNase treatment was required to rid samples of residual genomic DNA contamination. 17 µL of eluted RNA was added to a 20 µL final volume reaction of Turbo DNA-Free kit DNase (Thermo Fisher AM1907) according to the manufacturer's protocol. All ddPCR experiments had a “NoRT” control which tested negative for target amplification.

### DNA/RNA quantification

Concentration, 260/280, and 260/230 values were determined using a NanoDrop ND-1000 Spectrophotometer (Thermo Scientific).

### Reverse transcription

For all ddPCR experiments, 500 ng of sample RNA was reverse transcribed using the SuperScript III First-Strand Synthesis System according to the manufacture's protocol. A total of 2 µL of the supplied random hexamers mix was used for all samples which were not being tested for the presence of a poly(A) tail. An amount of 1 µL of the supplied oligo (dT) was used for samples being tested for the presence of a poly(A) tail in nontreated Hep3B cells. Additionally, 1 µL of 2 µM gene-specific primer was used in a separate reaction as a control to ensure specific amplification of the eRNA transcript. Gene-specific RT primer: 5′-TAGCCTCCTGGAGCGATTTA-3′.

### Droplet digital PCR

cDNA from reverse transcription reactions (500 ng input RNA) was diluted in the following ways. Detection of eRNA: no sample dilution was performed. CEBPA detection: HeLa samples received no dilution; Jurkat samples were diluted 1:5; Human Adipose RNA was diluted 1:20; Hep3B, HepG2, and HL-60 samples were diluted 1:100. GAPDH detection: Samples diluted 1:100 except Human Adipose RNA, which was diluted 1:20. CEBPG detection: Samples diluted 1:100. Albumin detection: Samples diluted 1:100. TBCB and B2M detection: All samples diluted 1:100. In all cases, 25 µL of the final sample dilutions were used to generate droplets for ddPCR performed using a QX200 (BioRad) and performed in multiplex, when applicable. Normalization was performed using GAPDH for all samples according to (Coulter 2018) with the exception that theoretical cDNA GAPDH input amounts in some cases, due to dilution differences, differed from theoretical cDNA input amounts of a given target and were adjusted accordingly. GAPDH did not differ significantly between control and treatment groups in all gapmer-based and saRNA-based experiments. For dCas9-VPR-based experiments, fold-change of GAPDH never exceeded −1.3 (far lower absolute difference than the observed changes in targets >4.0). Droplet detection analysis was performed using the associated QuantaSoft software (BioRad). The following ddPCR Expression Probe Assays were purchased from Biorad and used according to the manufacturer's protocol: CEBPA (fam) dHsaCPE5192369, GAPDH (hex) dHsaCPE503159, and Albumin (hex) dHsaCPE5056095, B2M (fam) dHsaCPE5053100, TBCB (fam) dHsaCPE5051586. Custom CRED9 (fam) and CEBPG (hex) PrimeTime taqman probe and primers were purchased from IDT: eRNA-Probe: /56-FAM/TTCAGCTCC/ZEN/ATGCCCAGTGGTC/3IABkFQ/ eRNA- R-qPCR: tctgaaggcactgtggtctgtc; eRNA-F-qPCR: cactgtgagccagggttctgt; CEBPG-Probe:/5HEX/C GAGAGAGG/ZEN/AACAACATGGCTGTGA/3IABkFQ/; CEBPG-For: CAAAAAGAGTTCG CCCATGGA; CEBPG-Rev: CTGCAGTGTGTCTTGTGCTTTC.

### ASO transfection

An amount of 150–200 nM final concentration of CRED9 targeting LNA-Gapmer or negative control was reverse transfected into HepG2 or Hep3b using 1.1 µL Lipofectamine RNAiMax (Thermo Fisher). Oligo-lipofectamine complexes were formed in reduced serum Opti-MEM media (Thermo Fisher). Cells were resuspended in complete RPMI media and ∼1.1 × 10^5^ cells were added to a 24-well plate containing the oligo-lipofectamine complexes in a final volume of 500 µL. Media was aspirated 6 h later followed by addition of fresh complete RPMI media at 1mL final volume. Cells were harvested 18 h after the change of media (i.e., 6 h treatment + 18 h incubation thereafter) using 300 µL TRIzol reagent followed by RNA isolation. Antisense LNA gapmers were designed using Exiqon's (now Qiagen) design tool and purchased from Qiagen. “*” Denotes phosphorothioate modified backbones. Negative Control A, cat# 339515 LG00000002-DDA. CtrlA: 5′-A*A*C*A*C*G*T*C*T*A*T*A*C*G*C-3′; Gap4: 5′-A*T*A*G*A*C*T*C*A*T*T*C*A*C*T*G-3′; Gap6: 5′-G*G*G*A*G*G*A*G*G*T*A*A*G*A*T*T-3′.

### Plasmid transfection

Plasmids harboring CRISPR guide RNA sequences and a plasmid coding for a CRISPR-VPR fusion protein were complexed with Lipofectamine 3000 (Thermo Fisher) and Opti-MEM according to the manufacturer's protocol. Plasmid-lipofectamine complexes were transfected into HeLa cells grown to ∼85% confluence in a 24-well plate at concentrations of 150 ng each (pair of guide sequence plasmids at 300 ng final pooled concentration) and 200 ng (SP-dCas9-VPR). Combinations of plasmids included a pair of control guide RNAs that target either firefly luciferase or a mitochondrial genome target sequence, a pair that target upstream of the TSS of CEBPA, or a pair that target upstream of the TSS of CRED9. CRISPRa guide RNAs were designed using CRISPR-era (Liu et al. 2015) (http://crispr-era.stanford.edu/). SP-dCas9-VPR (Addgene Plasmid #63798); PsiCHECK-1 with spCas9 scaffold for sgRNA expression of a mitochondrial DNA sequence sgRNA, used as (-) control, PsiCHECK-1 with spCas9 scaffold for sgRNA expression of firefly luciferase targeting sgRNA, used as : (-) control; pKLV-U6gRNA-EF(BbsI)-PGKpuro2ABFP (Addgene Plasmid #62348) with the following sequences cloned into the scaffold region: eCEB-act-sg6-F: 5′-CACCGAGAACAAGTATTCTCACTG-3′; eCEB-act-sg6-R: 5′-AAACCAGTGAGAATA CTTGTTCTC-3′; eCEB-act-sg2-F: 5′-CACCGTCGTGGTAGCATTCCTGGCA-3′; eCEB-act-sg2-R: 5′-AAACTGCCAGGAATGCTACCACGAC-3′; CEBPA promoter sgRNA: Cebpa-SG-act-2-F; 5′-CACCGGAG TATCCCGAGGCTGCACG-3′; Cebpa-SG-act-2-R: 5′-AAACCGTGCAGCCTCGGGATACTCC-3′; Cebpa-SG-act-5-F:5′-CACCGTGCAGCCTCGGGATACTCCT-3′; Cebpa-SG-act-5-R: 5′-AAACAGGAGTATCCC GAGGCTGCAC-3′. Plasmids from selected colonies were amplified in DH10b *E. coli* and purified by NucleoSpin Plasmid Mini kit for plasmid DNA 740588.50 (Machery-Nagel). Sequencing was performed by Eurofins Scientific.

### Nontransfected samples

HepG2, Hep3B, and HeLa cells were seeded at ∼8.0 × 10^4^ cells/well in 24-well plates and allowed to grow to 85% to 90% confluence before being harvested using 300 mL TRIzol reagent. HL-60 and Jurkat were harvested by collecting ∼1.5 × 10^5^ cells by centrifugation at 300*g* for 5 min, removal of supernatant, and addition of 300 mL TRIzol reagent.

### Genome browser tracks

Tracks were loaded and imaged from the UCSC genome browser (GRCh37/hg19) from the Genome Reference Consortium.

### Track credits

H3K27ac tracks from Roadmap Epigenomics Project: http://www.roadmapepigenomics.org/ (Bernstein et al. 2010). FANTOM5 consortium: Total counts of CAGE reads (Hon et al. 2017). ENCODE tracks: All ENCODE tracks (The ENCODE Project Consortium 2012; Wang et al. 2012; Davis et al. 2018). ChIP tracks HepG2 and Liver: FOXA1, FOXA2, CEBPB, HNF4A (The ENCODE Project Consortium 2012; Wang et al. 2012; Davis et al. 2018). ChIP tracks HepG2, Hela-S3, NB4: Pol-II, Pol-II S2, P300, CEBPA (Euskirchen et al. 2007; Rozowsky et al. 2009). H3K27ac: HepG2 and Hela-S3 (Ram et al. 2011). Vertebrate Multiz Alignment & Conservation: ENCODE (Davis et al. 2018) and Multiz (Blanchette et al. 2004). DNA Methylation by Reduced Representation Bisulfite Seq: ENCODE/HudsonAlpha (Meissner et al. 2008). Multiple cell line histone modification: H3K4me1, H3K4me3, H3K27ac (Ernst et al. 2011). Unibind Tracks: https://unibind.uio.no/ (Gheorghe et al. 2019).

### RNA-seq data

NX reads—http://www.proteinatlas.org (Uhlen et al. 2017); TMP reads—https://www.ebi.ac.uk/gxa/home (Petryszak et al. 2014).

### Short activating RNA (saRNA)

An amount of 30 nM final concentration of saRNA AW1-51 or scrambled control was reverse transfected into HepG2, Hep3B, or HeLa cell using 1.0 µL Lipofectamine RNAiMax (Thermo Fisher) according to the manufacturer's protocol. Oligo-lipofectamine complexes were formed in reduced serum Opti-MEM media (Thermo Fisher). Cells were resuspended in complete RPMI media and ∼1.1 × 10^5^ cells were added to a 24-well plate containing the oligo-lipofectamine complexes at a final volume of 500 µL. Media was aspirated 6 h later followed by addition of fresh complete RPMI media at 1 ml final volume. Cells were harvested 42 h after the change of media (i.e., 6 h treatment + 42 h incubation thereafter) using 300 µL TRIzol reagent (Thermo Fisher) followed by RNA isolation. Genomic DNA for bisulfite sequencing was isolated using NucleoSpin Tissue Mini kit for DNA from cells and tissue #740952.50 (Macherey-Nagel). AW1-51: Guide: 5′-GCGGUCAUUGUCACUGGUCUU-3′; Passenger: 5′-GACCAGUGACAAUGACCGCUU-3′. Scrambled Control: 5′-UCUACUGUCACUCAGUAGUUU-3′; 5′-ACUACUGAGUGACAGUAGAUU-3′.

### Fluorescent western blot

Approximately 3.5 × 10^5^ Hep3B or HepG2 cells were reverse transfected in six-well plates with 3.3 µL Lipofectamine RNAimax (Thermo Fisher) and a final concentration of 200 nM ctrlA or Gap4 LNA gapmers in Opti-MEM media (Thermo Fisher). Transfection media was changed with complete RPMI media after 6 h of incubation. Cells were harvested 18 h after the media change (i.e., 24 h after initial treatment). Cells were first washed two times with ice cold PBS followed by the addition of 300 µL ice cold RIPA lysis buffer #89901 (Thermo Fisher) supplemented with cOmplete, EDTA-free Protease Inhibitor Cocktail (Millipore Sigma) according to the manufacturer's protocol. Cells were allowed to lyse for 15 min with agitation at 4°C prior to collection. Samples were snap-frozen and stored at −80°C. Samples were allowed to thaw on ice prior to spinning down at 12,000 rpm on a tabletop centrifuge. Lysate was transferred away from pellet to a Qiashredder column (Qiagen) and spun according to the manufacturer's protocol. Protein was quantified using a Pierce BCA protein assay kit (Thermo Fisher) and analyzed on a SpectraMax iD3 multiplate reader (Molecular Devices). Lysates were reduced at 90°C in Pierce Lane Marker Reducing Sample Buffer (5×) #39000 (Thermo Fisher) for 5 min. An amount of 15 µg of each reduced sample lysate was added to a 12.5% SDS-PAGE gel in addition to PageRuler Prestained NIR protein ladder #26635 (Thermo Fisher). Protein was wet transferred to fluorescent membrane from the AzureSpectra Fluorescent Western blotting kit #AC2191 (Azure Biosystems). Membranes were first stained with Revert 700 Total Protein Stain for Western Blot Normalization #926-11010 (LI-COR) and imaged on a Sapphire Biomolecular Imager (Azure Biosystems). The image of stained protein was used for total protein normalization across all lanes. Membranes were then blocked in TBST with 5% milk for 15 min at RT followed by Azure Biosystems’ guidelines for blocking, washing, and antibody incubation. Recombinant anti-CEBPA antibody (EP709Y) (ab40764) diluted 1:1000. NIR Fluorescently labeled secondary antibody: Goat-anti-rabbit 800 #AC2134 (Azure Biosystems) diluted 1:10,000. Florescent images were captured on a Sapphire Biomolecular imager and subsequently analyzed using Azurespot software (Azure Biosystems).

### H3K27ac ChIP-qPCR

HepB3 cells were grown to ∼75%–80% confluency in six-well plates and transfected with ASO CtrlA (*n* = 2) or CRED9-speicfic GAP4 (*n* = 2) at 200 nM final concentration. Cells were incubated for 6 h prior to media replacement and then for an additional 18 h. Fixation was achieved by direct addition of formaldehyde to media (1% final concentration) for 10 min and quenched with 0.125 M glycine. Cells were collected and washed with ice-cold PBS as described by the Active Motif ChIP-IT High Sensitivity kit manual (Active Motif, cat# 43040) and sent to Active Motif Services to be processed for ChIP-qPCR. In brief, chromatin was isolated by the addition of lysis buffer, followed by disruption with a Dounce homogenizer. Lysates were sonicated and the DNA sheared to an average length of 300–500 bp. Genomic DNA (Input) was prepared by treating aliquots of chromatin with RNase, proteinase K and heat for decrosslinking, followed by ethanol precipitation. Pellets were resuspended and the resulting DNA was quantified on a NanoDrop spectrophotometer. Extrapolation to the original chromatin volume allowed quantitation of the total chromatin yield. An aliquot of chromatin (30 µg) was precleared with protein A agarose beads (Invitrogen). Genomic DNA regions of interest were isolated using 4 µg of antibody against H3K27Ac (Active Motif, cat# 39133). Complexes were washed, eluted from the beads with SDS buffer, and subjected to RNase and proteinase K treatment. Crosslinks were reversed by incubation overnight at 65°C, and ChIP DNA was purified by phenol–chloroform extraction and ethanol precipitation. qPCR reactions were carried out in triplicate on specific genomic regions using SYBR Green Supermix (Bio-Rad, cat# 170-8882) on a CFX Connect Real Time PCR system. The qPCR assay was performed using primer pairs against the CEBPA +9 kb enhancer region, positive control primer pairs that amplify known H3K27 acetylation sites within the *GAPDH* and *ACTB* promoters (Active Motif, cat# 71003/71005 and 71004/71006), as well as a negative control primer pair that amplifies a region in a gene desert on chromosome 12 (Active Motif, cat# 71001). The resulting signals were normalized for primer efficiency by carrying out qPCR for each primer pair using Input DNA.

### Statistical analysis

For ddPCR experiments, statistical analysis and graphs were generated using Prism-Graphpad (GraphPad Software). Error bars are represented as standard deviations. *P*-values were generated by unpaired *t*-tests of control vs. treatment groups. For western blot experiments, statistical analysis is reported as coefficients of variation (CV) of the fold change for each replicate measurement according to Licor's Quantitative Western Blot Replication Protocol (https://www.licor.com/documents/7bd2dev0rfjofad7dr1zf85wip4g4it3). CV = (std of fold change for replicates/mean fold change of replicates) × 100%. Fold changes which were greater than double the CV were considered valid.

## SUPPLEMENTAL MATERIAL

Supplemental material is available for this article.

## Supplementary Material

Supplemental Material
