# Supplementary material for: CRED9: a differentially expressed elncRNA regulates expression of transcription factor CEBPA
Source: RNA. 2021 Aug;27(8):891–906. doi: 10.1261/rna.078752.121 (PMC8284328; doi:10.1261/rna.078752.121)
Supplement: Supplemental Material [file supp_27_8_891__DC1.html]

CRED9: a differentially expressed elncRNA regulates expression of transcription factor CEBPA — Supplemental Material 

# CRED9: a differentially expressed elncRNA regulates expression of transcription factor CEBPA

## Supplemental Material

- Supplemental\_Data\_7\_2\_5RACE\_M13R\_JT2737\_2.zip
- Supplemental\_Data\_16\_1\_5RACE\_M13R\_JT2739\_4.zip
- Supplemental\_Data13\_2\_5RACE\_M13R\_JT2738\_3.zip
- Supplemental\_Material.xlsx
